# Thanks to Our Reviewers*, The Plant Genome*, 2019

**DOI:** 10.1002/tpg2.20026

**Published:** 2020-05-12

It is a great pleasure to thank the volunteer reviewers, without whom we could not publish *The Plant Genome*. Despite the demands of their everyday duties, the individuals listed below took time to assist the Editorial Board by reviewing one or more manuscripts. They demonstrate professionalism in the best sense. For their anonymous, unpaid efforts, all CSSA members owe them thanks. Every effort was made to compile an accurate list from the records kept by Editorial Board members. If we have omitted a name, we extend our apologies as well as our thanks.

Acosta, Juan

Adhikari, Laxman, Kansas State Univ., United States

Akdemir, Deniz

Akhunov, Eduard, Kansas State Univ., United States

Alvarez Prado, Santiago, IFEVA, Argentina

Arya, L., Natl. Bur. Plant Genet. Resources, India

Assmann, Sarah, Penn State, United States

Balint‐Kurti, Peter, USDA, United States

Balint‐Kurti, Peter, USDA, United States

Bartholome, Jerome

Bayer, Micha, The James Hutton Institute, United Kingdom of Great Britain and Northern Ireland

Bayer, Philipp, Univ. of Western Australia, Australia

Bernardo, Rex, Univ. of Minnesota, United States

Blanco, Antonio, Univ. of Bari, Italy

Blischak, Paul, Univ. of Arizona, United States

Bohn, Martin, Univ. of Illinois at Urbana‐Champaign, United States

Brown, Patrick, Univ. of California Davis, United States

Brummer, Edward, Univ. of California, Davis, United States

Busta, Lucas, Ctr. Plant Sci. Innovat., United States

Butt‐Wilmsmeyer, Carrie

Cameron, John, United States

Carrigan, Lori, United States

Ceoloni, Carla, Nature and Energy Univ. of Tuscia Viterbo Italy

Chebotarov, Dmytro, International Rice Research Institute, Philippines

Chen, Jianli, Univ. of Idaho, United States

Chen, Gengshen, China

Chen, Charles, Oklahoma State Univ., United States

Chen, Liqing

Chen, Lailiang

Clark, Lindsay, Univ. of Illinois

Cobb, Joshua, International Rice Research Institute, Philippines

Crain, Jared, Kansas State Univ., United States

Das, Shouvik, Indian Agricultural Research Institute, India

Datta, Sourav, Indian Inst. Sci. Educ., India

DellaPenna, Dean, Michigan State Univ.

Deng, Dewayne, USDA‐ARS, United States

Diepenbrock, Christine, Univ. of California, Davis, United States

Dinglasan, Eric, Cornell Univ. College of Agriculture and Life Sciences, United States

Duke, Stephen E., USDA‐ARS

Edwards, Jacqueline, Agriculture Victoria Services Pty Ltd, Australia

Endelman, Jeffrey, Univ. of Wisconsin, United States

Evans, Kate, Washington State Univ, United States

Falque, Matthieu, INRA, France

Fedak, George, Canada

Fellers, John, USDA‐ARS, United States

Fondevilla, Sara

Fotopoulos, Vasileios, Cyprus Univ. of Technology, Cyprus

Fritsche‐Neto, Roberto, Univ. de Sao Paulo Escola Superior de Agricultura Luiz de Queiroz, Brazil

Fukuta, Yoshimichi, JIRCAS Tropical Agriculture Research Front, Japan

Gage, Joseph, United States

Garland‐Campbell, Kimberly, USDA‐ARS, United States

Gaut, Brandon S., Univ California Irvine, United States

Gleason, Cynthia, Washington State Univ.

Goodwin, Paul, Univ. of Guelph, Canada

Haberer, Georg, Helmholtz Zentrum Munchen Deutsches Forschungszentrum fur Umwelt und Gesundheit, Germany

Han, Dejun, China

Harris, Neil, Univ. of Alberta, Canada

Himmelbauer, Heinz, Univ. of Natural Resources and Life Sciences Vienna, Austria

Ho, Julie, Forage Genetics International, United States

Howard, Reka, Univ. of Nebraska‐Lincoln, United States

Huang, Xuehui

Huggins, Trevis, USDA‐ARS, United States

Incognito, Salvador, Univ. Nacional de Lomas de Zamora Facultad de Ciencias Agrarias, Argentina

Isidro Sanchez, Julio, Univ. College Dublin, Ireland

Jacobson, Amy, Corteva Agriscience, United States

Jamann, Tiffany, Univ. of Illinois at Urbana‐Champaign, United States

Jarquin, Diego, Univ. of Nebraska‐Lincoln, United States

Jayakodi, Murukarthick, IPK‐Gatersleben, Germany

Jiang, Yong, Leibniz‐Institut fur Pflanzengenetik und Kulturpflanzenforschung Gatersleben, Germany

Jones, Elizabeth, Cornell Univ., United States

Juvik, John, United States

Kagale, Sateesh

Kamal, Nadia, Helmholtz Center Munich German Research Center for Environmental Health, Germany

Kantar, Michael, Univ. of Hawaii at Manoa, United States

Kim, Jan, Univ. of Hertfordshire, United Kingdom of Great Britain and Northern Ireland

Klein, Stephanie, Pennsylvania State Univ., United States

Klos, Kathy, USDA Agricultural Research Service, United States

Knox, Ron, Canada

Kolmer, James, USDA‐ARS Cereal Disease Laboratory, United States

Komatsuda, Takao, NIAS, Japan

Kuhlmann, Markus, Leibniz Inst. Plant Genet., Germany

Kumar, Satish, Plant and Food, NZ, New Zealand

Lado, Bettina, Univ. de la Republica, Uruguay

Laperche, Anne

Lawrence‐Dill, Carolyn, Iowa State Univ., United States

Leandro, Leonor, Iowa State Univ., United States

Li, Ying‐hui, Institute of Crop Science, China

Li, Xuehui, Noble Foundation, United States

Li, Chengdao, Murdoch Univ., Australia

Li, Mingjun, China

Liang, Zhikai, Univ. of Minnesota Twin Cities, United States

Liesche, Johannes

Liu, Zhaohui, North Dakota State Univ., United States

Liu, Liezhao

Liu, Ji‐hong, Huazhong Agricultural Univ., China

Liu, Aizhong, Chinese Academy of Science, United States

Lu, Fei

Lucretti, Sergio, ENEA Casaccia Research Centre, Italy

Luo, Lily

Ma, Qing‐Hua, Chinese Academy of Forestry Research Institute of Forestry, China

Ma, Jianxin, Purdue Univ., United States

Ma, Xiong‐Feng, Chinese Acad. Agr. Sci., United States

MacLean, Dan, Sainsbury Laboratory, United Kingdom of Great Britain and Northern Ireland

Maccaferri, Marco, Univ. of Bologna, Italy

Mascher, Martin, IPK Gatersleben, Germany

Mastrangelo, Anna Maria, Council Agr. Res., Italy

Matias, Filipe, Universidade de Sao Paulo Escola Superior de Agricultura Luiz de Queiroz Departamento de Genetica, Brazil

May, Gregory, National Center for Genomic Resources, United States

McCartney, Curt, Agriculture and Agri‐Food Canada, Canada

McClean, Phillip, North Dakota State Univ., United States

Meyers, Blake, Donald Danforth Plant Science Center, United States

Mian, Rouf, USDA

Miskovic, Ljubisa, EPFL, Switzerland

Mora, Freddy

Moradi, Parviz, AREEO, United States

N'Diaye, Amidou, Univ. of Saskatchewan, Canada

Newman, Jonathan, Univ. of Guelph College of Biological Science, Canada

Oladzad, Atena, North Dakota State Univ., United States

Pepper, Alan, Texas A&M Univ., United States

Petitot, Anne‐Sophie, UMR‐Interactions Plants Microorganisms Environment

Qiu, Li‐juan, Chinese Academy of Agricultural Sciences, China

Raatz, Bodo, Ciat, Colombia

Raman, Harsh, Graham Centre for Agric Innovation, Australia

Riechers, Dean

Romero, Irene, United States

Rowan, Beth, Univ. of California Davis, United States

Shi, Ainong, Univ. of Arkansas, United States

Said, Mahmoud, Institute of Experimental Botany AS CR, Czech Republic

Sallam, Ahmad, Univ. of Minnesota, United States

Salvi, Silvio, Univ. of Bologna, Italy

Sanhueza, Dayan, Univ Andres Bello, United States

Santantonio, Nicolas, Cornell Univ., United States

Sari, Eshan, National Research Council Canada, Canada

Savic, Jasna, Univ. of Belgrade Faculty of Agriculture, Serbia

Schmid, Karl

Schnable, James, Univ. of Nebraska, Lincoln, United States

Schoen, Chris‐Carolin, Germany

Sehgal, Deepmala, CIMMYT, Mexico

Sha, Tang, China

Sha, Xueyan, Univ. of Arkansas

Slaughter, Lindsey C., Univ. Kentucky, United States

Soares, Jose Serio, Universidade Estadual de Campinas, Brazil

Song, Qijian, United States

Sood, Shilpa, The Climate Corporation, United States

Spannagl, Manuel, Helmholtz Center Munich, Germany

Stetter, Markus, United States

Studholme, David

Sunkar, Ramanjulu, Oklahoma State Univ., United States

Swanton, Clarence, Univ. of Guelph, Canada

Thirugnanasambandam, Prathima Perumal, The Univ. of Queensland, United States

Tian, Bin, Kansas State Univ., United States

Tinker, Nicholas, AAFC, Canada

Torkamaneh, Davoud, Université Laval, Canada

Tricker, Penny, The Univ. of Adelaide, Australia

Usadel, Björn

Van Deynze, Allen, Univ. of California, United States

Van Sanford, David, Univ. of Kentucky, United States

Varotto, Serena, Univ. of Padova, Italy

Vi‐Cao, Tuong

Vitezica, Zulma

Vuong, Tri, Univ. of Missouri, United States

Walia, Harkamal, Univ. of Nebraska System, United States

Walkowiak, Sean

Wan, Xiangyuan, Univ. of Science and Technology Beijing, China

Wang, Zhiqiang, Chinese Acad. Agr. Sci., China

Wang, Rui, Univ. Idaho, United States

Wang, Dechun, Michigan State Univ., United States

Wang, Bo, Huazhong Agricultural Univ. College of Plant Science and Technology

Wang, Jiankang

Wang, Kan, Iowa State Univ., United States

Watson, Amy, Univ. of Queensland, Australia

Wiersma, Andrew, Michigan State Univ., United States

Wolfe, Marnin, Cornell Univ., United States

Wu, Yongrui

Wu, Jing, Chinese Acad. Agr. Sci., China

Xiao, Guanghui, Shaanxi Normal Univ. College of Life Sciences, United States

Xing, Anqi, Michigan State Univ., United States

Xing, Shiyan, Coll Shandong Agr. Univ, China

Xiong, Aisheng, Nanjing Agricultural Univ., China

Xu, Yunbi, CIMMYT, Mexico

Xu, Wei, Univ. Chinese Acad. Sci., China

Yan, Huihuang

Yan, Yueming, Capital Normal Univ., China

Yerka, Melinda, Univ. of Nevada Reno, United States

Yokoi, Shuji, Osaka Prefecture Univ., Japan

Yuan, Youlu, Chinese Acad. Agr. Sci., China

Yuan, Lingling, United States

Zakaria, Kehel, ICARDA

Zeng, Wenfang, Chinese Acad. Agr. Sci., China

Zhang, Xuecai, International Maize and Wheat Improvement Center (CIMMYT), Mexico

Zheng, Xiaoming, China

